# Significant Rewiring of the Transcriptome and Proteome of an *Escherichia coli* Strain Harboring a Tailored Exogenous Global Regulator IrrE

**DOI:** 10.1371/journal.pone.0037126

**Published:** 2012-07-05

**Authors:** Tingjian Chen, Jianqing Wang, Lingli Zeng, Rizong Li, Jicong Li, Yilu Chen, Zhanglin Lin

**Affiliations:** 1 Department of Chemical Engineering, Tsinghua University, Beijing, China; 2 College of Biotechnology and Pharmaceutical Engineering, Nanjing University of Technology, Nanjing, China; Center for Genomic Regulation, Spain

## Abstract

Cell reprogramming for microorganisms via engineered or artificial transcription factors and RNA polymerase mutants has presented a powerful tool for eliciting complex traits that are practically useful particularly for industrial strains, and for understanding at the global level the regulatory network of gene transcription. We previously further showed that an exogenous global regulator IrrE (derived from the extreme radiation-resistant bacterium *Deinococcus radiodurans*) can be tailored to confer *Escherichia coli* (*E. coli*) with significantly enhanced tolerances to different stresses. In this work, based on comparative transcriptomic and proteomic analyses of the representative strains E1 and E0, harboring the ethanol-tolerant IrrE mutant E1 and the ethanol-intolerant wild type IrrE, respectively, we found that the transcriptome and proteome of *E. coli* were extensively rewired by the tailored IrrE protein. Overall, 1196 genes (or approximately 27% of *E. coli* genes) were significantly altered at the transcriptomic level, including notably genes in the nitrate-nitrite-nitric oxide (NO) pathway, and genes for non-coding RNAs. The proteomic profile revealed significant up- or downregulation of several proteins associated with syntheses of the cell membrane and cell wall. Analyses of the intracellular NO level and cell growth under reduced temperature supported a close correlation between NO and ethanol tolerance, and also suggests a role for membrane fluidity. The significantly different omic profiles of strain E1 indicate that IrrE functions as a global regulator in *E. coli*, and that IrrE may be evolved for other cellular tolerances. In this sense, it will provide synthetic biology with a practical and evolvable regulatory “part” that operates at a higher level of complexity than local regulators. This work also suggests a possibility of introducing and engineering other exogenous global regulators to rewire the genomes of microorganism cells.

## Introduction

In recent years, it has been shown that the transcriptomes of microorganisms can be altered by artificial transcription factors, mutants of innate transcription factors and RNA polymerase, and an exogenous global regulator (IrrE) and its mutants, leading to significantly altered cellular phenotypes [1]–[7]. This suggests a clearly complex but amendable regulatory network for a given genome.

IrrE is derived from the radiation-tolerant bacterium *Deinococcus radiodurans* (*D. radiodurans*). It has been described as a global regulator responsible for the exceptional DNA repair capability for the microbe [8], possibly by activating RecA and PprA instead of directly participating in this process [9]–[11]. It also seems that the regulatory range of IrrE does not limit to DNA repair, since other pathways, including transcriptional regulation, stress responses, energy metabolism, signal transduction, and protein turnover were also significantly altered compared to an IrrE-deficient mutant of *D. radiodurans* [12]. However, the exact molecular mechanism of IrrE remains to be elucidated. It is unclear whether it directly binds DNA, other transcription factors, or RNA polymerase. The crystal structure of *Deinococcus Deserti* IrrE, a homology of the IrrE used in this study, reveals three domains in IrrE, including the N-terminal domain with a mono zinc metallopeptidase fold, the middle domain with a helix-turn-helix (HTH) motif, and the C-terminal domain sharing high structural similarity with the GAF “sensor” domain commonly present in signal-transducing proteins [13]. Although the HTH motif is widely found in transcriptional factors, the DNA-binding motif in IrrE has a rather unusual location between the N- and C-terminal domains, and there has been no direct evidence to support that this domain binds DNA. One hypothesis is that IrrE functions at least in part by proteolytic cleavage of “transcriptional messengers” by interacting with small molecules [13].

The wild type *D. radiodurans* IrrE was also found to be able to enhance certain tolerances in non-native hosts, for example towards radiation, osmotic stress, heat stress and oxidative stress in *Escherichia coli* (*E. coli*) [14]–[16] and salt stress in *Brassica napus* [16]. Previously we further established a number of laboratory-evolved variants of IrrE [3], that can reprogram and confer *E. coli* cells with much enhanced tolerances toward ethanol, butanol and acetate [3], as well as inhibitors in lignocellulosic hydrolysates (Wang, *et al.*, unpublished). The variants were selected from a library of randomly mutated *irrE* genes introduced into *E. coli*, which is widely used to produce biochemicals and biofuels. One of these variants, E1, confers *E. coli* with markedly enhanced tolerance toward ethanol compared with the control strain. There are four mutations (M19V, T42S, V100A and E275G) in E1, all of which are on the surface [3], and none occurs at the reported critical sites for IrrE (E119, H122, Y196 and H260) [13]. In this work, using the ethanol-tolerant strain E1 (the DH5α strain containing the IrrE mutant E1) as a model, we performed transcriptomic and proteomic analyses, and compared the profiles with those of the original strain E0 expressing the wild type IrrE, a strain that is not ethanol tolerant. Strikingly, approximately 27% of *E. coli* genes (1196 genes) were significantly altered at the transcriptomic level. This number is much greater compared with that for engineered sigma factor variants (around 100 genes) [2], but comparable with that for RNA polymerase mutants (around 1000 genes) [4]. These differentially transcribed genes in strain E1 included those in the nitrate-nitrite-nitric oxide (NO) pathway, and dozens for non-coding RNAs (ncRNAs). Interestingly, a number of other pathways that were only recently discovered to be associated with cellular stress responses or tolerances were also altered significantly in strain E1, including tryptophan transport and metabolism, iron transport and utilization, and oxidative phosphorylation [17]–[20]. Classical pathways that are already well known to correlate with cellular tolerances, such as transport and metabolism pathways of glycerol and trehalose [21], were also found in strain E1. The proteomic analysis was less informative, but complemented the transcriptomic data. The roles of some of these pathways in cellular tolerance to ethanol were validated by experimental assays. The efficient reprogramming of *E. coli* cells via tailored IrrE has implications for fundamental understanding of the regulatory network for the transcriptome and proteome of microorganisms. It also expands the toolbox for synthetic biology by providing an evolvable regulatory “part” that functions at a higher level of complexity [21].

## Results

### Transcriptome Profiles of the Strains E1 and E0

To directly analyze the alterations at the transcriptomic and proteomic levels in *E. coli* achieved by the engineered IrrE mutant E1 that confers ethanol tolerance while the wild type IrrE does not, we compared the global transcriptome and proteome between *E. coli* DH5α strains E1 and E0, harboring the *irrE* mutant E1 and the wild type *irrE* gene, respectively, under ethanol stress. The transcriptomes of these two strains exposed to ethanol stress were assayed using DNA microarrays. The results of the DNA microarray assays showed that the transcription of 688 genes was upregulated by more than 2-fold (log_2_ ratio >1) in strain E1 compared with that in strain E0, while 508 genes were downregulated in strain E1 to less than 0.5-fold (log_2_ ratio <−1) of that in strain E0. Overall, approximately 27% of the total number of genes in *E. coli* showed significantly different levels of transcription due to the introduction of the IrrE mutant E1, which is greater than the extent of genes differentially transcribed because of mutations in the sigma factor RpoD using the global transcription machinery engineering (gTME) approach (around 100 genes were differentially expressed) [2]. These results indicate that IrrE mutant E1 can reprogram the global transcription of the *E. coli* genome.

**Figure 1:**
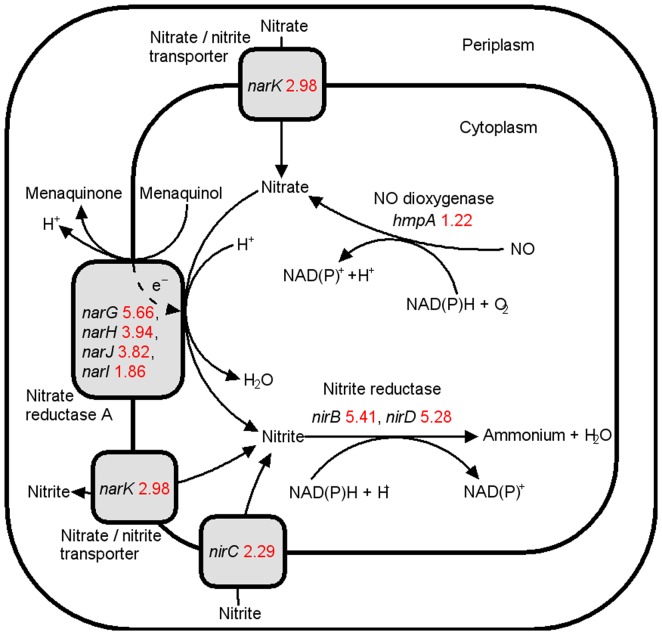
Differentially expressed genes associated with the metabolism and transport of NO, nitrate and nitrite. The number after each gene is the Log_2_ value (fold change in E1 compared with E0). Red values: upregulated in E1; dashed arrows: direction of electron flow.

**Figure 2:**
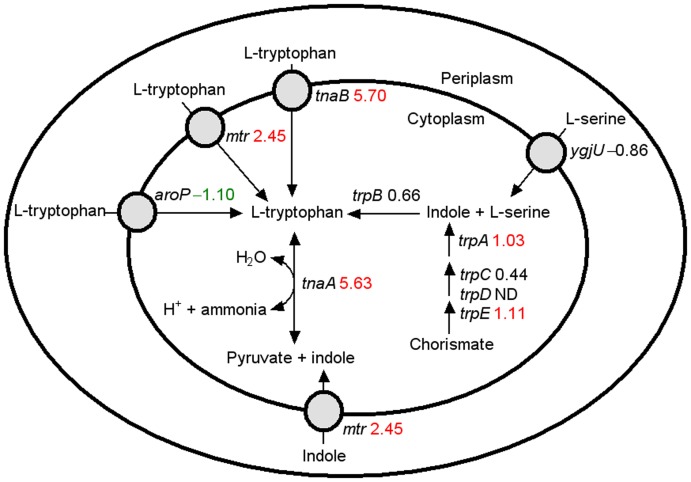
Differentially expressed genes associated with tryptophan metabolism and transport. The number after each gene is the Log_2_ value (fold change in E1 compared with E0). Red values: upregulated in E1; green values: downregulated in E1; black values: no difference in expression.

The genes and pathways showing the most significant changes at the transcriptional level in strain E1 are summarized below (**Figures 1**,**2** **,** **3**, and **S1,S2**; **Tables 1** **,** **2**, and **S1**). The pathways that showed the greatest upregulation in strain E1 included the transport and metabolism of nitric oxide, nitrate and nitrite, tryptophan transport and metabolism, oxidative phosphorylation, and glycerol transport and metabolism. The genes showing the greatest downregulation in strain E1 included those involved in iron transport and utilization. Dataset S1 summarizes the other genes showing significant changes at the transcriptional level.

**Figure 3:**
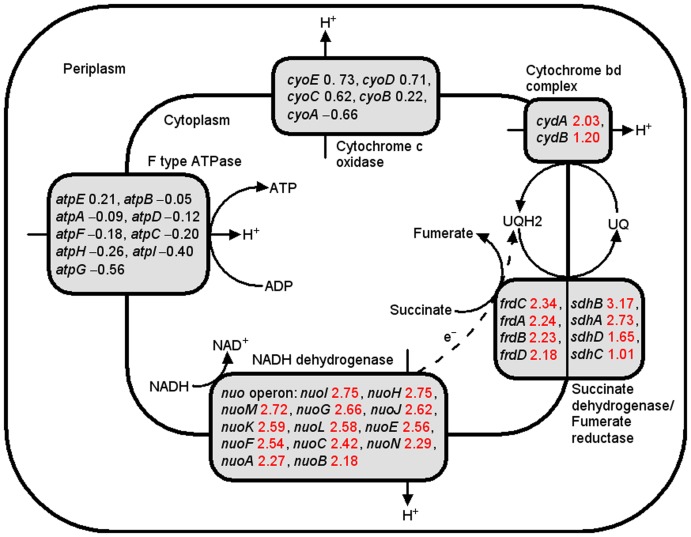
Differentially expressed genes associated with oxidative phosphorylation. The number after each gene is the Log_2_ value (fold change in E1 compared with E0). Red values: upregulated in E1; black values: no difference in expression; dashed arrows: direction of electron flow.

**Table 1:** Differentially expressed genes associated with iron transport and metabolism.

Gene	Functional description	Fold change in strain E1 relative to E0 (Log_2_ ratio)
*bfr*	Bacterioferritin, iron storage and detoxification protein	1.81
*feoA*	Ferrous iron transport protein A	1.78
*feoB*	Fused ferrous iron transporter, protein B: GTP-binding protein	1.73
*fepD*	Iron-enterobactin transporter membrane protein	–1.16
*b1451*	Predicted iron outer membrane transporter	–1.29
*fhuB*	Iron-hydroxamate transporter permease subunit	–1.44
*fepG*	Iron-enterobactin transporter permease	–1.53
*fhuD*	Iron-hydroxamate transporter substrate-binding subunit	–1.65
*fhuA*	Ferrichrome outer membrane transporter	–1.74
*fepC*	Iron-enterobactin transporter ATP-binding protein	–2.06
*fecE*	Iron-dicitrate transporter subunit	–2.16
*fepB*	Iron-enterobactin transporter periplasmic binding protein	–2.37
*fecD*	Iron-dicitrate transporter subunit	–2.42
*fhuF*	Ferric iron reductase involved in ferric hydroximate transport	–2.58
*fes*	Enterobactin/ferric enterobactin esterase	–2.91
*fecB*	Iron-dicitrate transporter subunit	–3.09
*fecI*	RNA polymerase, sigma 19 factor	–3.15
*fecR*	Transmembrane signal transducer for ferric citrate transport	–3.46
*fecA*	Ferric citrate outer membrane transporter	–3.57
*fepA*	Outer membrane receptor FepA/iron-enterobactin outer membrane transporter	–3.70
*fhuE*	Ferric-rhodotorulic acid outer membrane transporter	–3.90
*cirA*	Colicin I receptor/ferric iron-catecholate outer membrane transporter	–4.48
*fiu*	Catecholate siderophore receptor Fiu/predicted iron outer membrane transporter	–5.07

**Table 2:** Differentially expressed non-coding RNAs.

Gene	Length (nt)	Fold change in strain E1relative to E0 (Log_2_ ratio)	Gene	Length (nt)	Fold change in strain E1 relative to E0 (Log_2_ ratio)
*ryeE*	86	4.48	*ssrS*	183	1.17
*ryeC*	143	3.15	*dicF*	53	1.09
*isrA*	158	3.10	*rydB*	68	1.06
*rygA*	88	2.61	*rdlC*	68	–1.02
*rygB*	76	2.47	*rdlB*	66	–1.03
*ryeA*	249	2.11	*ffs*	114	–1.04
*rygC*	140	2.00	*oxyS*	110	–1.57
*csrB*	360	1.95	*sraB*	169	–1.60
*ryhA*	108	1.39	*ryhB*	90	–2.09
*csrC*	245	1.23	*spf*	109	–2.10
*micF*	93	1.21	*rtT*	171	–2.61
*ryjA*	140	1.19			

#### (1) NO, nitrate and nitrite transport and metabolism

As shown in **Figure 1**, NO dioxygenase transforms NO into nitrate, which is then reduced by nitrate reductase to nitrite, followed by reduction by nitrite reductase to ammonium. Under ethanol stress, the transcriptional levels of nitrate reductase, nitrite reductase, nitrate/nitrite transporter and NO dioxygenase were upregulated in strain E1 (**Table S1**). NO plays a role as a stress signal in *E. coli*, but is harmful to the cell at high concentrations [22]–[25]. The upregulation of genes in this pathway is likely to help reduce the intracellular concentrations of NO, nitrate and nitrite following exposure to ethanol stress, and thus reduce cellular damage caused by NO. This assumption is partially confirmed by the results of the NO assay, which are described below.

#### (2) Tryptophan transport, metabolism and regulation

As shown in **Table S1**, many genes in this pathway were upregulated under ethanol stress, particularly genes related to tryptophan transport and degradation, such as *tnaB*, *mtr* and *tnaA* (**Figure 2**). This finding is consistent with several recent reports. For example, it has been shown that supplementing the culture medium with tryptophan increased *E. coli* tolerance to ethanol [17] and that the gene expression of *tnaA* was associated with *E. coli* tolerance toward isobutanol [26]. More interestingly, it was recently found that indole is an important signal molecule in the *E. coli* stress response, by switching on the cellular anti-oxidant system, the expression of genes involved in NO detoxification, and the expression of efflux pumps to clear harmful molecules [5], [27]. As shown in **Figure 2**, the significant upregulation of *tnaB*, *mtr* and *tnaA* genes in strain E1 will likely lead to an increase in the intracellular indole concentration. Additionally, the tryptophan repressor (TrpR) binding protein WrbA, which is thought to regulate the tryptophan operon and has an NAD(P)H:quinone oxidoreductase activity [28], was significantly upregulated in strain E1 (**Table S1**). This protein is also implicated in *E. coli* response to oxidative stress [28].

#### (3) Iron transport and utilization

As shown in **Table 1**, the transcription of 23 genes involved in iron transport and utilization was significantly altered in strain E1. Notably, many of the genes associated with the transport of ferric iron were downregulated, while those associated with the transport of ferrous iron, including *feoA* and *feoB*, were upregulated. It was recently reported that genes involved in the transport of ferric iron were downregulated in a butanol-tolerant *E. coli* mutant [18] while overexpression of *feoA* increased the tolerance of *E. coli* to butanol [19]. Taken together, these findings suggest that as iron in both the reduced and oxidized states are crucial for the activities of many intracellular proteins, the availability of ferrous and ferric irons may affect many cellular metabolic processes, and thus alter the cellular tolerance.

#### (4) Oxidative phosphorylation

As shown in **Table S1**, several subunits of cytochrome d terminal oxidase, fumarate reductase, succinate dehydrogenase and NADH dehydrogenase were upregulated in strain E1 exposed to ethanol stress. As shown in **Figure 3**, upregulation of these genes will increase the proton gradient across the cell membrane, and counteract the loss of the proton-driving force across the cell membrane, because of the increased membrane fluidity in the presence of alcohol [29]. It has also been reported that the *nuo* operon, *sdhCDAB* and *cydAB* genes were upregulated in *E. coli* exposed to butanol stress [20]. Lee *et al*. showed that *sdhCDAB* were also significantly upregulated in a butanol-tolerant mutant harboring an artificial transcription factor, and that overexpression of these proteins enhanced cellular tolerance toward butanol [18]. It has also been postulated that, since these proteins are membrane proteins, their upregulation can change the fluidity of the cell membrane, and hence increase cellular tolerance toward alcohols [18].

#### (5) ROS detoxification

As shown in **Table S1**, the transcription of several enzymes related to ROS degradation was significantly upregulated in strain E1, including superoxide dismutases *sodB* and *sodC*, and hydroperoxidases *katE* and *katG*. These results are consistent with the lower ROS level in strain E1 exposed to ethanol stress, as we have previously reported [3].

#### (6) Ribosome proteins

As shown in **Table S1**, many genes associated with ribosome were differential expressed in strain E1 compared with strain E0, which may be related to the changes in translation activity in strain E1 [30]. Rutherford *et al*. recently also reported changes in the transcription of genes encoding ribosome proteins in cells exposed to butanol stress [20].

#### (7) Other significantly altered pathways and proteins

As shown in **Table S1**, other significantly altered pathways or proteins included: glycerol metabolism and transport, trehalose metabolism, glycogen metabolism, phosphoenolpyruvate/sugar phosphotransferase (PTS) system, outer membrane proteins, ABC transporters, stress response proteins and chaperon proteins. Among these, glycerol is well-known to enhance with stress tolerance in many microorganisms [21]. Under ethanol stress, many genes related to glycerol metabolism and transport were upregulated in strain E1 (**Table S1** and **Figure S1**). For example, *glpC* showed the largest fold-increase in expression of all of the genes upregulated in strain E1. The GlpC protein is the subunit that anchors glycerol-3-phosphate dehydrogenase to the cell membrane in *E. coli*. It is then not surprising that, as described below, the addition of glycerol to the culture medium significantly enhanced the tolerance of strain E0 to ethanol. Trehalose is also strongly associated with cellular tolerance toward osmotic, salt, nutrient, oxidative and ethanol stresses [31]–[34]. As shown in **Table S1**, genes involved in trehalose metabolism were all upregulated in strain E1. The PTS system controls the use of carbon sources in the cell. Of all of the outer membrane genes, *ompW* showed the greatest increase in expression in strain E1. OmpW was reported to be involved in the transport of small hydrophobic molecules across the outer membrane, and to protect cells against damage induced by toxic substances [35], [36]. The sigma factor RpoS is one of the main cellular stress-response proteins, and controls the expression of many genes involved in the stress response [37]. In strain E1, the upregulation of RpoS appears to be directly related to the changes in the transcription of genes in the trehalose metabolism pathway, and to the change in that of *glgS* which regulates glycogen metabolism.

#### (8) Non-coding RNAs

As shown in **Table 2**, the transcription of many genes for non-coding RNAs was altered in strain E1. One of these genes, *micF*, is the anti-sense RNA for *ompF*, and its upregulation will reduce the expression level of OmpF [38]. OmpF is a channel protein that controls the transport of hydrophilic small molecules across the outer membrane. Downregulation of this protein was found to enhance cellular tolerances to toxic compounds [39]. *oxyS* is a sensor of intracellular oxidative stress and is upregulated by oxidative stress [40]. In strain E1, the transcription of *oxyS* was downregulated, which was probably caused by the lower intracellular ROS level in strain E1 [3]. *ryhB* regulates iron utilization by promoting degradation of mRNAs for proteins involved in iron utilization, particularly *bfr* mRNA [41]. *ryhB* can also stimulate degradation of *sdhCDAB* mRNA in the oxidative phosphorylation pathway and *sodB* mRNA in the ROS detoxification pathway [42]. Therefore, downregulation of *ryhB* expression in strain E1 should increase the mRNA levels of *bfr*, *sdhCDAB* and *sodB*. This is consistent with the transcriptional changes detected in these genes (**Tables 1** and **S1**). We also found that the transcription of aconitase AcnA and fumarase FumA, whose mRNA levels are reversely related to *ryhB* [43] was significantly upregulated in strain E1 (log_2_ ratios: 1.91 and 2.71, respectively, Dataset S1). *rygA* and *rygB* negatively regulate mRNAs of the outer membrane proteins OmpT, CirA, FecA and FepA [44]. Therefore, upregulation of *rygA* and *rygB* in strain E1 should lead to decreases in the mRNA levels of these genes and, in fact, we found decreased levels of these genes, as shown in **Tables 1** and **S1**. The importance of non-coding RNAs in the regulation of gene expression in bacteria is becoming increasingly clear in recent years [45], [46]. It has also been reported that non-coding RNAs are involved in the cellular responses to oxidative stress, acid shock and cell envelope stress generated by SDS and EDTA [40], [47]. Our work, however, represents the first reported evidence for the extensive role of non-coding RNAs in response to ethanol stress in *E. coli*.

### Proteome Profiles of the Strains E1 and E0

We next compared the proteomes of the strains E0 and E1 under ethanol stress, using two-dimensional (2D) gel electrophoresis and matrix-assisted laser desorption/ionization time of flight mass spectrometry (MALDI-TOF-MS). The results of 2D gel electrophoresis showed that 122 protein spots were upregulated in strain E1 by more than 4-fold (log_2_>2) compared with that in strain E0, while 120 protein spots were downregulated in strain E1 to less than 0.25-fold (log_2_<−2) of that in strain E0. A total of 62 protein spots showing the largest fold changes, which were well separated, were successfully identified by MALDI-TOF-MS, yielding 51 distinct proteins, since some of the proteins displayed multiple dots in the 2D gels. Of these, 22 proteins were upregulated and 29 downregulated in strain E1 compared with strain E0.

As shown in **Tables S2** and **S3**, the upregulated proteins included proteins associated with: (1) amino acid metabolism (tryptophan and lysine) (TnaA and DapA), (2) oxidative phosphorylation (SdhA, NuoF and PapA), (3) glycerol metabolism (GlpD), (4) the outer membrane (OmpW), (5) the ribosome and phenylalanine-tRNA synthetase (RplB, RplI and PheS), (6) sugar or sugar derivative metabolism (PfkA, Gnd, and KdgK), (7) tricarboxylic acid cycle (SucD), (8) nucleotide metabolism (PyrG and PurH), (9) fatty acid metabolism (AccC), (10) small organic acid metabolism (AckA), (11) cell membrane and cell wall synthesis (LpxA and KdsA), and (12) transcription (Rho), as well as (13) a putative protein (YcnE).

Among these proteins, TnaA, OmpW, GlpD, NuoF, SdhA, PfkA, KdgK and SucD were upregulated significantly at both the transcriptional and protein levels, while RplB, RplI, PheS, PyrG, AccC, PurH, AckA, LpxA and Rho were upregulated at the protein level, but downregulated at the transcriptional level (**Table S3**). Although the reason for this discrepancy is unclear, it should be note that IrrE has a postulated protease activity [3], [13], which might contribute to this observed discrepancy. In addition, some non-coding RNAs, such as *oxyS*, *spf*, *micF* and *ryhB*, might alter the proteomic profile by disturbing the translation process [48].

It is interesting to note that KdsA, which showed the greatest increase in expression of all proteins in strain E1, is a key enzyme in the biosynthesis of lipopolysaccharide [49], a key component of the outer membrane of Gram-negative bacteria. Meanwhile, LpxA, which was also significantly upregulated in strain E1, catalyzes the first reaction step in the biosynthesis of lipids [50], a major component of the cell membrane and cell wall.

As shown in **Table S2**, the downregulated proteins included proteins associated with (1) amino acid metabolism (serine, glycine and proline) (GlyA, GcvT, SerC and ProA), (2) oxidative phosphorylation (NuoC), (3) glycerol metabolism (GlpK and GapA), (4) the outer membrane (TolC, CirA, OmpT, OmpA, Fiu and OmpX), (5) an oligopeptide transporter protein (OppA), (6) translation (RpsA, ProS, GlyS, TrpS and FusA), (7) sugar or sugar derivative metabolism (Crr, TalB, PckA, TktA, MdoG, LpdA and AceE), (8) transcription (YhgF), (9) RNA degradation (Pnp) and (10) antibiotic resistance (Bla).

Among these proteins, GlyA, SerC, CirA, OmpT, Fiu, OmpX, RpsA, ProS, GlyS, FusA, AceE, YhgF and Pnp were downregulated significantly both at the transcriptional and protein levels, while GcvT, NuoC, GlpK, GapA, OppA and PckA were downregulated at the protein level, but upregulated at the transcriptional level (**Table S3**). As described above, the reason for this discrepancy is unclear.

Of the 51 proteins identified by MALDI-TOF-MS, changes in the expression of 28 were consistent with the corresponding changes at the transcriptional level, while 22 were not. One protein, YncE, was not detected in the DNA array assay. In comparing these data, it is also important to note the following. First, membrane proteins are often difficult to detect and identify by 2D gel electrophoresis and MALDI-TOF-MS [51], but many of the genes associated with ethanol tolerance in our transcriptome analysis were membrane proteins (for example, **Table S1** and **Figures 1** **,** **3**). Second, the non-coding RNAs, which are important factors in the stress response, as demonstrated in our study and in an earlier study [45], can only be analyzed using DNA microarrays. Third, different threshold values are often used to select differentially expressed genes in transcriptome and proteome analyses [52], [53]. In our study, these thresholds were |log_2_| >1, and |log_2_| >2, respectively. Finally, only 62 of 244 spots for differentially expressed proteins were chosen for identification with MALDI-TOF-MS.

### Effects of Ethanol Stress on Intracellular NO Concentrations

**Figure 4:**
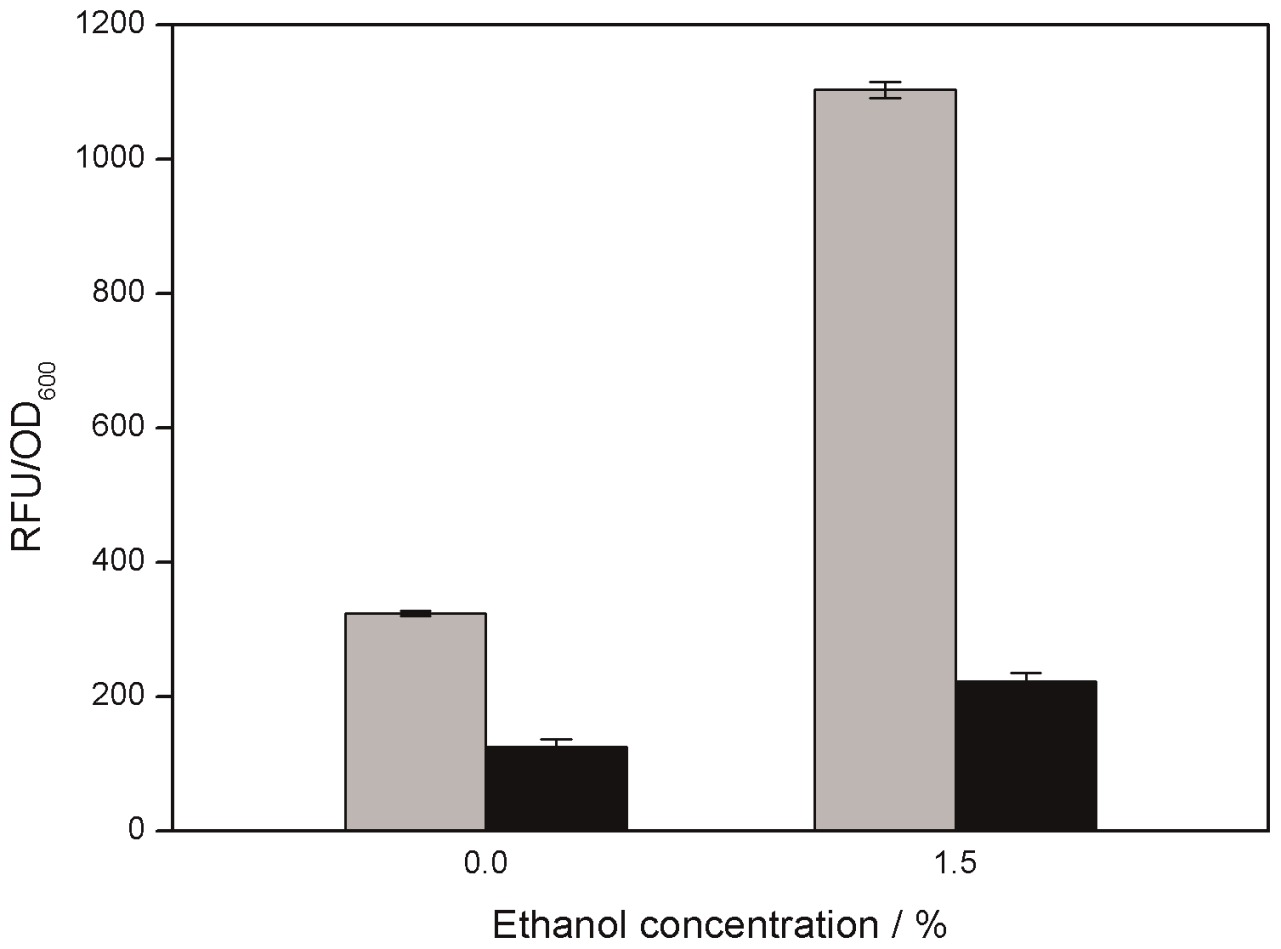
Comparison of intracellular NO concentrations. Relative fluorescence unit (RFU) per OD of cells of strains E0 (grey) and E1 (black) cultured in LB medium supplemented with 0% or 1.5% ethanol.

The transcriptomic analyses revealed that many genes associated with the metabolism and transport of NO, nitrate and nitrite were upregulated in strain E1 compared with that in strain E0 under ethanol stress. Therefore, we measured the NO concentrations in both strains under ethanol stress. As shown in **Figure 4**, the intracellular NO concentration in strain E0 increased significantly in response to ethanol stress, with only slight increases in strain E1. These results are consistent with the omics analyses.

### Effects of Glycerol Supplementation or Reduced Culture Temperature on Ethanol Tolerance

**Figure 5:**
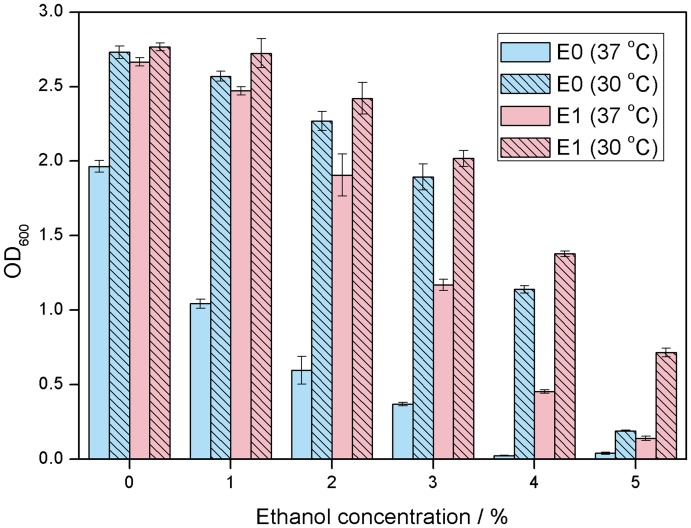
Effects of culture temperature on ethanol tolerance of strains E0 and E1. Values are OD_600_ values measured after culture at 30°C or 37°C for 23 hours.

Since the glycerol transport and metabolism pathway was significantly upregulated in strain E1, and glycerol is often found to be correlated with enhanced tolerances toward several stresses including organic solvent tolerance for *E. coli* [54], butanol tolerance for *Clostridium acetobutylicum* [55], osmotic tolerance for *Saccharomyces cerevisiae* [32]. Therefore, we tested the association between glycerol and cellular tolerance by measuring cell growths of strains E0 and E1 cultured in the presence of ethanol and 0.5% glycerol. The results (**Figure S3**) clearly showed that appropriate concentrations of glycerol can improve cellular tolerance to ethanol significantly, especially for strain E0. Many of the genes upregulated in strain E1 encode membrane proteins, including those involved in glycerol metabolism, oxidative phosphorylation, trehalose metabolism, as well as NO, nitrate and nitrite metabolism. Since the upregulation of these membrane proteins might affect the fluidity of the cell membrane, we tested the effect of reduced temperature culture (30°C) on ethanol tolerance of strains E0 and E1, as this condition decreases membrane fluidity [56], [57]. Indeed, as shown in **Figure 5**, the ethanol tolerance of both strains was significantly higher when cultured at 30°C than at 37°C. The fold change in OD_600_ for strain E1 ranged from 1.1-fold at 1% ethanol to 5.1-fold at 5% ethanol, and was even more pronounced for strain E0, ranging from 1.4-fold at 1% to 45.6-fold at 4%. These results suggest that the reduced membrane fluidity during culture at lower temperatures contributes to ethanol tolerance.

## Discussion

In this study, we show that the transcriptomic and proteomic profiles of *E. coli* strain E1 harboring the ethanol-tolerant global regulator mutant E1 were significantly different from those of the starting strain E0 containing the wild type IrrE, which does not confer ethanol tolerance. While the exact mechanism remains to be dissected, the results again suggest that the IrrE mutant E1 indeed can rewire the *E. coli* transcriptome and proteome. In particular, many genes associated with the transport and metabolism of NO, nitrate and nitrite, tryptophan, iron, and glycerol, as well as those associated with oxidative phosphorylation and non-coding RNAs were significantly up- or downregulated at the transcriptional level. At the protein level, several of the proteins in the aforementioned pathways, including TnaA, GlpD, OmpW, CirA, Fiu, SdhA and NuoF, were also significantly up- or downregulated. Moreover, the proteomic analyses revealed several other differentially expressed proteins that may contribute to ethanol tolerance, including outer membrane proteins and proteins associated with synthesis of cell membrane and cell wall. These findings at the transcriptional and protein levels were validated in part by intracellular NO assays, and by cell growth in response to reduced temperature culture. These results clearly indicate the extensive ability of the IrrE variant E1 in rewiring the *E. coli* genome, and provide us with new and more specific clues to enhance the cellular tolerances of *E. coli* to various stresses.

The ethanol-tolerant strain E1 was directly compared to the strain containing the wild type IrrE (strain E0) that is not ethanol tolerant, so that we could obtain features in strain E1 that confers the improved tolerance, and avoid other irrelevant factors introduced by the mere presence of the wild type IrrE in *E. coli* (*i.e.*, if strain E1 was compared to the strain containing the blank plasmid). Along this line, it is interesting to compare our data with those of *E. coli* harboring wild type IrrE in the absence of stress or under salt stress [7], [16]. Zhou *et al*. reported that in the absence of stress, about 365 genes were differentially transcribed in the presence versus the absence of wild type IrrE (at a lower threshold of log_2_>0.848 or <−0.848) [7]. Therefore, the altered transcriptomic profile in strain E1 (*vs* strain E0 harboring the wild type IrrE), extends significantly from that of the strain harboring the wild type IrrE (*vs E. coli* alone). Only several genes or pathways showed similar patterns of changes, which included the catalase gene *katE* involved in ROS detoxification, genes in the trehalose metabolism pathway, and the maltose transporter gene *malE*. It is this extended perturbation caused by the IrrE mutant E1 that confers ethanol tolerance in strain E1, since wild type IrrE alone does not [3]. Under salt stress (1 M NaCl), about 124 proteins were differentially expressed in the *E. coli* strain harboring wild type IrrE (at log_2_>1 or <−1). These proteins included tryptophan repressor binding protein, glycerol-degrading proteins, ribosome protein (RpsF), ROS detoxification proteins, several stress response proteins and protein kinases [16]. While none of these proteins were significantly altered in our proteomic study (where the threshold values were also more stringent at log_2_>2 or <−2), the trends for several of these proteins were similar to those in our transcriptomic results. These include the stress-responsive sigma factor RpoS, catalase KatE and TrpR-binding protein WrbA. Taken together, the changes in the transcriptome and proteome of strain E1 subjected to ethanol stress were generally different from those of *E. coli* harboring wild type IrrE and subjected to salt stress. These results suggest that the mechanism involved in cellular tolerance to ethanol differs from that for tolerance to salt stress. Therefore it is not surprising that, although wild type IrrE confers *E. coli* with greater salt tolerance, it does not confer higher ethanol tolerance [3].

The significantly different performances between the ethanol tolerant IrrE mutants obtained previously [3] (including E1 used in this study) and the wild type IrrE, suggest that IrrE has a promise to be an evolvable global regulator that can alter the cellular phenotypes of *E. coli* beyond ethanol stress, and perhaps beyond the host *E. coli*. The introduction and engineering of IrrE in *E. coli* represents an artificial horizontal gene transfer event in which an exogenous gene is transferred into a new host, and evolves to adapt to the new host and confers the host with new traits. This work suggests a vast possibility of introducing and tailoring other exogenous global regulators for reprogramming gene expression, which will no doubt expand the toolbox for synthetic biology. These regulators will present practical regulatory “parts” that operate at a much higher level of complexity, and will be useful for eliciting complex phenotypes that are otherwise difficult to improve by manipulating individual genes or pathways, for example, cellular tolerances of industrial microorganisms toward different stresses. Along this line, it is interesting to note that IrrE-like global regulators have only been identified in the genus *Deinococcus*. Further search for IrrE-like regulators in other microorganisms is thus warranted.

Compared with those approaches that reprogram the cells by engineered innate transcription factors, RNA polymerase, or artificial transcription factors, the IrrE (or IrrE like proteins) approach has several potential advantages. First, an exogenous global regulator like IrrE exists in the cell separately from the innate cellular transcription machinery, and thus would be presumably more amendable. Second, IrrE, which is likely to have different recognition sequences and regulation mechanisms from those of endogenous transcriptional factors, would extend the perturbation range of the transcriptional profiles. Finally, although the mechanism involved in IrrE regulation is still not well understood [13], it was postulated to have both transcriptional regulation and protease activities [13], and thus may provide a way to simultaneously alter the transcriptomic and proteomic profiles of the host cell.

## Materials and Methods

### Bacterial Strains and Culture Conditions

*E. coli* DH5α strains E1 and E0 were described previously [3]. Cells were grown at 37°C in Luria-Bertani (LB) medium containing 50 µg ml^–1^ ampicillin overnight, then diluted at a ratio of 1∶100 into LB medium containing 1.5% (v/v) ethanol and 50 µg ml^–1^ ampicillin, and grown at 37°C. The cells were harvested at the exponential phase, and then rapidly chilled and stored in liquid nitrogen until used for the DNA microarray assay and 2D gel electrophoresis. Each sample was prepared and analyzed in triplicate.

### DNA Microarray Analysis

DNA microarray analysis of the samples was carried out by CapitalBio (Beijing, China). Briefly, total RNA was isolated from the cells stored in liquid nitrogen using the TRIzol Reagent (Invitrogen Life Technologies, Carlsbad, CA, USA) and RNeasy MinElute Cleanup Kits (Qiagen, Valencia, CA, USA). *E. coli* genome 2.0 arrays (Affymetrix, Santa Clara, CA, USA) were used for hybridization, and arrays were run in triplicate with biological replicates to allow for statistical confidence for differential gene expression. The slides were scanned by a GeneChip Scanner 3000 (Affymetrix) and analyzed using GeneChip Operating Software Version 1.4 (Affymetrix). All the microarray data were deposited in the GEO database at the National Center for Biotechnology Information with accession number GSE30441.

### 2D gel Electrophoresis and Mass Spectrometric Analysis

2D gel electrophoresis and mass spectrometric analysis of the samples were carried out by the Beijing Proteome Research Center (Beijing, China), using an apparatus from Bio-Rad (Hercules, CA, USA). The differentially expressed protein dots were identified by MALDI-TOF-MS using an ABI-8000 mass spectrometer (ABI, Carlsbad, CA, USA).

### Intracellular NO Assay

Cells were grown at 37°C overnight in LB medium (supplemented with 50 µg ml^–1^ ampicillin) containing 0% or 1.5% ethanol, and then harvested. The cell pellets were washed and resuspended with 0.1 M sodium phosphate buffer (SPB) (pH 7.4). The intracellular NO levels were determined essentially as described [58], with a Spectra-Max M2 microplate reader (Molecular Devices, Sunnyvale, CA, USA).

### Tolerance Tests with Metabolite Supplementation

Cells were grown at 37°C in LB medium containing 50 µg ml^–1^ ampicillin overnight. The cultures were then diluted to an OD_600_ of 0.02 in LB medium containing 50 µg ml^–1^ ampicillin, as well as the indicated concentrations of metabolites and ethanol, and then grown at 37°C, or at 30°C to test tolerance at a reduced temperature. OD_600_ was monitored during growth.

## Supporting Information

Figure S1 **Differentially expressed genes associated with glycerol metabolism.** The number after each gene is the Log_2_ value (fold change in E1 compared with E0). Red values: upregulated in E1; black values: no difference in expression.
(TIF)

Figure S2 **Differentially expressed genes associated with glycogen metabolism.** The number after each gene is the Log_2_ value (fold change in E1 compared with E0). Red values: upregulated in E1.
(TIF)

Figure S3 **Effects of glycerol on the ethanol tolerance of strains E0 and E1.** Values are OD_600_ values measured after culture without or with 0.5% glycerol for 23 hours.
(TIF)

Table S1 **Significantly altered pathways or gene clusters in mutant E1.**
(DOC)

Table S2 **Identification of proteins differentially expressed in strain E1.**
(DOC)

Table S3 **Comparison of changes at the protein and transcriptional levels in strain E1 relative to strain E0.**
(DOC)

Dataset S1 **Summary of the differentially expressed genes in strain E1 relative to strain E0.**
(XLS)
