# Supplementary figures and images for: Significant Rewiring of the Transcriptome and Proteome of an Escherichia coli Strain Harboring a Tailored Exogenous Global Regulator IrrE
Source: PLoS One. 2012 Jul 5;7(7):e37126. doi: 10.1371/journal.pone.0037126 (PMC3390347; doi:10.1371/journal.pone.0037126)

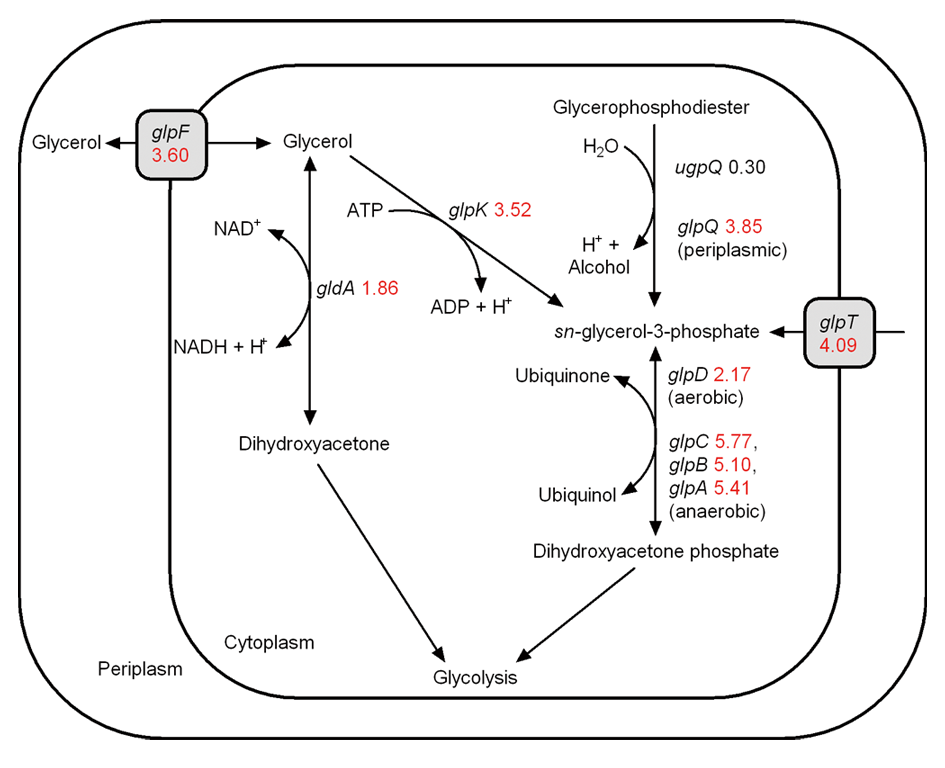

Supplement: Figure S1 — Differentially expressed genes associated with glycerol metabolism. The number after each gene is the Log2 value (fold change in E1 compared with E0). Red values: upregulated in E1; black values: no difference in expression. (TIF) [file pone.0037126.s001.tif]

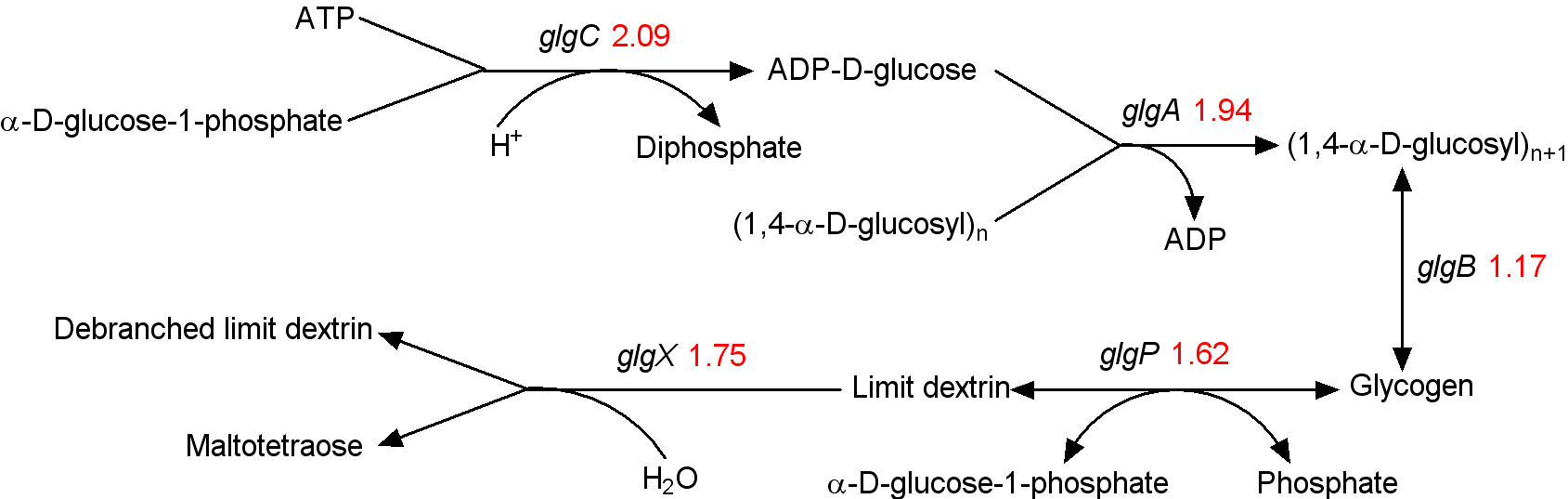

Supplement: Figure S2 — Differentially expressed genes associated with glycogen metabolism. The number after each gene is the Log2 value (fold change in E1 compared with E0). Red values: upregulated in E1. (TIF) [file pone.0037126.s002.tif]

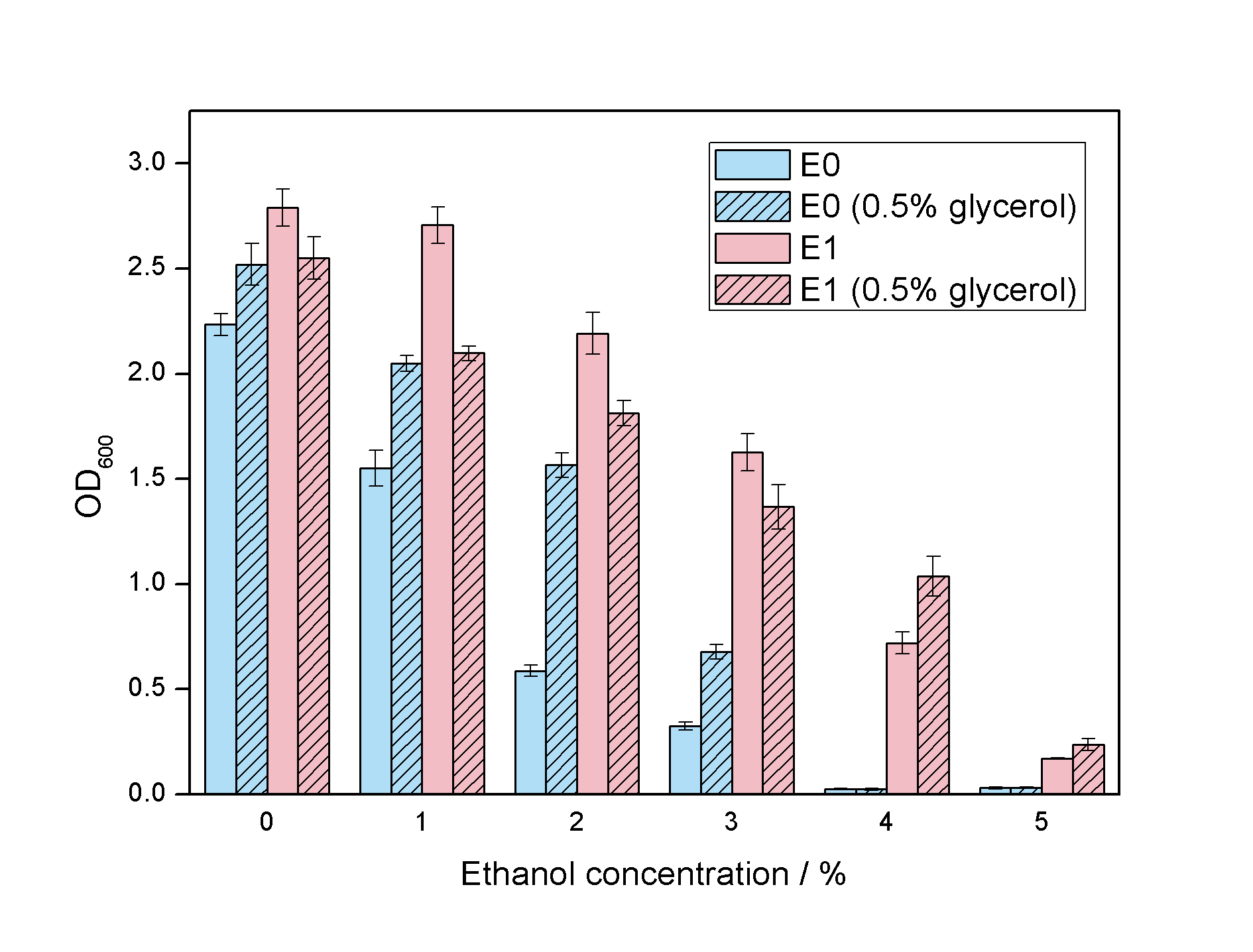

Supplement: Figure S3 — Effects of glycerol on the ethanol tolerance of strains E0 and E1. Values are OD600 values measured after culture without or with 0.5% glycerol for 23 hours. (TIF) [file pone.0037126.s003.tif]
