# Supplementary material for: Significant Rewiring of the Transcriptome and Proteome of an Escherichia coli Strain Harboring a Tailored Exogenous Global Regulator IrrE
Source: PLoS One. 2012 Jul 5;7(7):e37126. doi: 10.1371/journal.pone.0037126 (PMC3390347; doi:10.1371/journal.pone.0037126)
Supplement: Table S1 — Significantly altered pathways or gene clusters in mutant E1. (DOC) [file pone.0037126.s004.doc]

**Table S1.** Significantly altered pathways or gene clusters in mutant E1

| **Gene** | **Functional description** | **Fold change in strain E1 relative to E0 (Log_2_ ratio)** |
| --- | --- | --- |
| **Transport and metabolism of NO, nitrate and nitrite** | | |
| *narG* | Nitrate reductase 1, α subunit | 5.66 |
| *nirB* | Nitrite reductase (NAD(P)H) subunit | 5.41 |
| *nirD* | Nitrite reductase small subunit | 5.28 |
| *narH* | Nitrate reductase 1 β subunit | 3.94 |
| *narJ* | Nitrate reductase 1 δ subunit | 3.82 |
| *narK* | Nitrite extrusion protein | 2.98 |
| *nirC* | Nitrite transporter NirC | 2.29 |
| *narI* | Nitrate reductase 1 γ subunit | 1.86 |
| *hmpA* | NO dioxygenase | 1.22 |
| **Tryptophan transport and metabolism** | | |
| *tnaL* | Tryptophanase leader peptide | 5.70 |
| *tnaB* | Tryptophan permease TnaB | 5.70 |
| *tnaA* | Tryptophanase | 5.63 |
| *wrbA* | TrpR binding protein WrbA | 3.61 |
| *mtr* | Tryptophan permease | 2.45 |
| *trpL* | Trp operon leader peptide | 1.28 |
| *trpA* | Tryptophan synthase subunit α | 1.03 |
| *trpE* | Anthranilate synthase component I | 1.11 |
| **Oxidative phosphorylation** | | |
| *cydA* | Cytochrome d terminal oxidase, polypeptide subunit I | 2.03 |
| *cydB* | Cytochrome d terminal oxidase polypeptide subunit II | 1.20 |
| *frdC* | Fumarate reductase subunit C | 2.34 |
| *frdA* | Fumarate reductase flavoprotein subunit | 2.24 |
| *frdB* | Fumarate reductase iron-sulfur subunit | 2.23 |
| *frdD* | Fumarate reductase subunit D | 2.18 |
| *sdhB* | Succinate dehydrogenase iron-sulfur subunit | 3.17 |
| *sdhA* | Succinate dehydrogenase flavoprotein subunit | 2.73 |
| *sdhD* | Succinate dehydrogenase cytochrome b556 small membrane subunit | 1.65 |
| *sdhC* | Succinate dehydrogenase cytochrome b556 large membrane subunit | 1.01 |
| *nuoI* | NADH dehydrogenase subunit I | 2.75 |
| *nuoH* | NADH dehydrogenase subunit H | 2.75 |
| *nuoM* | NADH dehydrogenase subunit M | 2.72 |
| *nuoG* | NADH dehydrogenase subunit G | 2.66 |
| *nuoJ* | NADH dehydrogenase subunit J | 2.62 |
| *nuoK* | NADH dehydrogenase subunit K | 2.59 |
| *nuoL* | NADH dehydrogenase subunit L | 2.58 |
| *nuoE* | NADH dehydrogenase subunit E | 2.56 |
| *nuoF* | NADH dehydrogenase I subunit F | 2.54 |
| *nuoC* | Bifunctional NADH:ubiquinone oxidoreductase subunit C/D | 2.42 |
| *nuoN* | NADH dehydrogenase subunit N | 2.29 |
| *nuoA* | NADH dehydrogenase subunit A | 2.27 |
| *nuoB* | NADH dehydrogenase subunit B | 2.18 |
| **ROS clearance** | | |
| *sodB* | Superoxide dismutase | 3.96 |
| *sodC* | Superoxide dismutase | 1.56 |
| *katE* | Hydroperoxidase II | 1.70 |
| *katG* | Hydroperoxidase HPI(I) | 1.26 |
| **Glycerol transport and metabolism** | | |
| *glpC* | sn-glycerol-3-phosphate dehydrogenase (anaerobic), small subunit | 5.77 |
| *glpA* | sn-glycerol-3-phosphate dehydrogenase (anaerobic), large subunit, FAD/NAD(P)-binding | 5.41 |
| *glpB* | Anaerobic glycerol-3-phosphate dehydrogenase subunit B | 5.10 |
| *glpT* | sn-glycerol-3-phosphate transporter | 4.09 |
| *glpQ* | Glycerophosphodiester phosphodiesterase | 3.85 |
| *glpF* | Glycerol facilitator protein | 3.60 |
| *glpK* | Glycerol kinase | 3.52 |
| *glpD* | Glycerol-3-phosphate dehydrogenase | 2.17 |
| *gldA* | Glycerol dehydrogenase | 1.86 |
| **Glycogen metabolism** | | |
| *glgS* | Glycogen synthesis protein GlgS | 5.24 |
| *glgC* | Glucose-1-phosphate adenylyltransferase | 2.09 |
| *glgA* | Glycogen synthase | 1.94 |
| *glgX* | Glycogen debranching enzyme | 1.75 |
| *glgP* | Glycogen phosphorylase | 1.62 |
| *glgB* | Glycogen branching enzyme | 1.17 |
| **Trehalose metabolism** | | |
| *treB* | Trehalose (maltose)-specific PTS system components IIBC | 1.81 |
| *treA* | Periplasmic trehalase | 1.79 |
| *treC* | Trehalose-6-phosphate hydrolase | 1.59 |
| *otsA* | Trehalose-6-phosphate synthase | 1.74 |
| *otsB* | Trehalose-6-phosphate phosphatase | 1.61 |
| **PTS system** | | |
| *fruB* | Bifunctional fructose-specific PTS IIA/HPr protein | 4.24 |
| *gatB* | Galactitol-specific PTS system component IIB | 2.89 |
| *gatA* | Galactitol-specific PTS system component IIA | 2.61 |
| *agaV* | N-acetylgalactosamine-specific PTS system transporter subunit IIB | 2.08 |
| *treB* | Trehalose(maltose)-specific PTS system components IIBC | 1.81 |
| *srlA* | Glucitol/sorbitol-specific enzyme IIC component of PTS | 1.65 |
| *agaW* | N-acetylgalactosamine-specific PTS system enzyme IIC component | 1.48 |
| *srlE* | PTS system, glucitol/sorbitol-specific IIBC component | 1.23 |
| *yadI* | Putative PTS enzyme II B component | 1.20 |
| *srlB* | Glucitol/sorbitol-specific PTS system component IIA | 1.00 |
| b1198 | Fused predicted dihydroxyacetone-specific PTS enzymes: HPr component/EI component | –1.46 |
| *ptsG* | Glucose-specific PTS system IIBC components | –2.70 |
| **Outer membrane proteins** | | |
| *ompW* | Outer membrane protein W | 3.82 |
| *blc* | Outer membrane lipoprotein Blc | 2.61 |
| *lamB* | Maltose outer membrane porin (maltoporin) | 2.48 |
| b1964 | Putative outer membrane protein | 2.04 |
| *ompC* | Outer membrane porin protein C | 1.42 |
| *slp* | Slp / outer membrane protein induced after carbon starvation | 1.18 |
| *yhjG* | Hypothetical protein / predicted outer membrane biogenesis protein | 1.12 |
| *spr* | Putative outer membrane lipoprotein | 1.08 |
| b2250 | Hypothetical protein / predicted outer membrane porin protein | –1.06 |
| *ompX* | Outer membrane protein X | –1.09 |
| b1451 | Predicted iron outer membrane transporter | –1.29 |
| b1505 | Outer membrane usher protein fimD precursor | –1.78 |
| *ompT* | Outer membrane protease | –2.41 |
| *wza* | Hypothetical protein / putative polysaccharide export protein | –2.67 |
| *yiaD* | Putative outer membrane lipoprotein | –2.94 |
| **ABC transporters** | | |
| b1440 | ABC transporter periplasmic-binding protein | 4.60 |
| *ybaE* | Predicted transporter subunit: periplasmic-binding component of ABC superfamily | 4.14 |
| b1442 | ABC transporter permease | 3.21 |
| *malE* | Maltose ABC transporter periplasmic protein | 3.2 |
| *ytfQ* | Predicted sugar transporter subunit: periplasmic-binding component of ABC superfamily | 2.77 |
| *mglA* | Fused methyl-galactoside transporter subunits of ABC superfamily: ATP-binding components | 2.72 |
| *rbsA* | Fused D-ribose transporter subunits of ABC superfamily: ATP-binding components | 2.50 |
| *rbsC* | Ribose ABC transporter permease protein | 2.49 |
| *yhiH* | Putative ATP-binding component of a transport system | 2.38 |
| *glnH* | Glutamine ABC transporter periplasmic protein | 2.32 |
| *xylG* | Fused D-xylose transporter subunits of ABC superfamily: ATP-binding components | 2.30 |
| *ydcV* | ABC transporter permease | 2.19 |
| *yhhJ* | Predicted transporter subunit: membrane component of ABC superfamily | 2.11 |
| b0830 | Predicted peptide transporter subunit: periplasmic-binding component of ABC superfamily | 2.02 |
| *mtlA* | Mannitol-specific PTS system enzyme IIABC components | 1.98 |
| b3020 | Predicted transporter subunit: periplasmic-binding component of ABC superfamily | 1.90 |
| b0831 | Predicted peptide transporter subunit: membrane component of ABC superfamily | 1.75 |
| *yjfF* | Inner membrane ABC transporter permease protein YjfF | 1.73 |
| b0832 | Predicted peptide transporter subunit: membrane component of ABC superfamily | 1.54 |
| *ytfR* | Predicted sugar transporter subunit: ATP-binding component of ABC superfamily | 1.22 |
| b0829 | Fused predicted peptide transport subunits of ABC superfamily: ATP-binding components | 1.07 |
| *araG* | Fused L-arabinose transporter subunits of ABC superfamily: ATP-binding components | 1.06 |
| *yrbF* | Putative ABC transporter ATP-binding protein YrbF | –1.23 |
| *yrbE* | Predicted toluene transporter subunit: membrane component of ABC superfamily | –1.23 |
| *yojI* | Fused predicted multidrug transport subunits of ABC superfamily: membrane component/ATP-binding component | –1.34 |
| *fhuB* | Fused iron-hydroxamate transporter subunits of ABC superfamily: membrane components | –1.44 |
| b1682 | Component of SufBCD complex, ATP-binding component of ABC superfamily | –1.95 |
| *pstS* | Phosphate ABC transporter periplasmic substrate-binding protein PstS | –2.26 |
| **Stress response proteins** | | |
| *ybdQ* | Universal stress protein UP12 | 3.01 |
| *yhiO* | Universal stress protein UspB | 2.78 |
| *ydaA* | Universal stress protein UspE | 2.37 |
| *yjbJ* | Putative stress-response protein | 1.89 |
| *yecG* | Universal stress protein UspC | 1.87 |
| *yiiT* | Universal stress protein UspD | 1.73 |
| *osmC* | Osmotically inducible, stress-inducible membrane protein | 1.30 |
| *uspA* | Universal stress protein A | 1.16 |
| *rpoS* | RNA polymerase sigma factor RpoS | 1.08 |
| b3913 | Periplasmic protein combats stress | –1.16 |
| b3914 | Periplasmic protein combats stress | –1.23 |
| **Chaperone proteins** | | |
| *yedU* | Chaperone protein HchA | 3.57 |
| *clpB* | Protein disaggregation chaperone | 1.98 |
| *cbpA* | Curved DNA-binding protein CbpA | 1.93 |
| *hypC* | Hydrogenase assembly chaperone | 1.85 |
| *yccD* | Modulator of CbpA co-chaperone | 1.82 |
| *hscA* | Chaperone protein HscA | –1.17 |
| *yegD* | Putative chaperone | –1.35 |
| b2527 | dnaJ-like molecular chaperone specific for IscU | –2.22 |
| **Genes associated with the ribosome** | | |
| *rmf* | Ribosome modulation factor | 3.94 |
| *rimL* | Ribosomal-protein-L7/L12-serine acetyltransferase | 2.65 |
| *yhiH* | Fused ribosome-associated ATPase: ATP-binding protein | 2.38 |
| *rpsV* | 30S ribosomal subunit S22 | 2.22 |
| *yfiA* | Cold shock protein associated with 30S ribosomal subunit | 1.52 |
| *yjbC* | 23S rRNA U2604 pseudouridine synthase | 1.27 |
| *rimJ* | Ribosomal-protein-S5-alanine N-acetyltransferase | 1.14 |
| *yfgB* | 23S rRNA m(2)A2503 methyltransferase, SAM-dependen | –1.00 |
| *yihA* | Ribosome biogenesis GTP-binding protein YsxC | –1.01 |
| b4371 | 16S ribosomal RNA m2G1207 methyltransferase | –1.01 |
| *rpsI* | 30S ribosomal protein S9 | –1.01 |
| *rimI* | Acetylase for 30S ribosomal subunit protein S18 | –1.02 |
| b1822 | 23S rRNA m(1)G745 methyltransferase | –1.03 |
| b2608 | 16S rRNA-processing protein RimM | –1.03 |
| *yebU* | 16S rRNA m(5)C1407 methyltransferase, SAM-dependent | –1.06 |
| *rplM* | 50S ribosomal protein L13 | –1.06 |
| *rplD* | 50S ribosomal protein L4 | –1.07 |
| b1086 | 23S rRNA pseudouridylate synthase C | –1.09 |
| *rplV* | 50S ribosomal protein L22 | –1.10 |
| *rpmA* | 50S ribosomal protein L27 | –1.13 |
| *rplQ* | 50S ribosomal protein L17 | –1.16 |
| *rpmB* | 50S ribosomal protein L28 | –1.17 |
| *yibK* | Putative tRNA/rRNA methyltransferase YibK | –1.18 |
| *gidB* | 16S rRNA methyltransferase GidB | –1.20 |
| *rplW* | 50S ribosomal protein L23 | –1.27 |
| *rpsU* | 30S ribosomal protein S21 | –1.28 |
| *ybeA* | rRNA large subunit methyltransferase | –1.34 |
| *rpsJ* | 30S ribosomal protein S10 | –1.36 |
| *rplC* | 50S ribosomal protein L3 | –1.37 |
| *rpsO* | 30S ribosomal protein S15 | –1.45 |
| *rplY* | 50S ribosomal protein L25 | –1.56 |
| *ygjO* | 23S rRNA mG1835 methyltransferase, SAM-dependent | –1.86 |
| *rpsT* | 30S ribosomal protein S20 | –2.09 |
