# Supplementary material for: Significant Rewiring of the Transcriptome and Proteome of an Escherichia coli Strain Harboring a Tailored Exogenous Global Regulator IrrE
Source: PLoS One. 2012 Jul 5;7(7):e37126. doi: 10.1371/journal.pone.0037126 (PMC3390347; doi:10.1371/journal.pone.0037126)
Supplement: Table S2 — Identification of proteins differentially expressed in strain E1. (DOC) [file pone.0037126.s005.doc]

**Table S2.** Identification of proteins differentially expressed in strain E1*

| **Protein** | **Functional description** | **Fold change in strain E1 relative to E0 (Log_2_ ratio)** |
| --- | --- | --- |
| **Proteins upregulated** | | |
| KdsA | 2-dehydro-3-deoxyphosphooctonate aldolase | 7.42 |
| AccC | Acetyl-CoA carboxylase biotin carboxylase subunit | 6.64 |
| PfkA | 6-phosphofructokinase | 5.38 |
| NuoF | NADH:ubiquinone oxidoreductase, chain F | 5.08 |
| Gnd | 6-phosphogluconate dehydrogenase | 5.06 |
| PurH | Bifunctional phosphoribosylaminoimidazolecarboxamide formyltransferase/IMP cyclohydrolase | 5.06 |
| Rho | Transcription termination factor Rho | 5.02 |
| PyrG | CTP synthetase | 4.64 |
| DapA | Dihydrodipicolinate synthase | 4.64 |
| PheS | Phenylalanine-tRNA synthetase, α subunit | 4.52 |
| TnaA | Tryptophanase | 4.40 |
| KdgK | 2-dehydro-3-deoxygluconokinase | 4.32 |
| GlpD | sn-glycerol-3-phosphate dehydrogenase, aerobic | 4.32 |
| SdhA | Succinate dehydrogenase flavoprotein subunit | 4.32 |
| YncE | Conserved protein | 4.32 |
| LpxA | UDP-N-acetylglucosamine acyltransferase | 3.39 |
| AckA | Acetate kinase | 3.32 |
| SucD | Succinyl-CoA synthetase subunit α | 3.24 |
| OmpW | Outer membrane protein W | 2.56 |
| RplB | 50S ribosomal protein L2 | 2.19 |
| RplI | 50S ribosomal protein L9 | 2.06 |
| PapA | Unnamed protein product | 1.02 |
| **Proteins downregulated** | | |
| GlpK | Glycerol kinase | –0.07 |
| PckA | Phosphoenolpyruvate carboxykinase | –0.89 |
| Bla | β-lactamase | –2.18 |
| OmpA | Outer membrane protein A | –2.25 |
| Crr | Glucose-specific enzyme IIA component of PTS | –3.38 |
| LpdA | Dihydrolipoamide dehydrogenase | –3.51 |
| TrpS | Tryptophanyl-tRNA synthetase | –3.57 |
| OmpX | Outer membrane protease [Plasmid F] | –3.64 |
| Pnp | Polynucleotide phosphorylase / polyadenylase | –3.68 |
| OmpT | Outer membrane protease | –3.85 |
| SerC | Phosphoserine aminotransferase | –3.90 |
| RpsA | 30S ribosomal protein S1 | –4.11 |
| Fiu | Predicted iron outer membrane transporter | –4.11 |
| AceE | Pyruvate dehydrogenase subunit E1 | –4.27 |
| TktA | Transketolase | –4.29 |
| OppA | Oligopeptide transporter subunit | –4.47 |
| GapA | Glyceraldehyde-3-phosphate dehydrogenase | –4.64 |
| YhgF | Predicted transcriptional accessory protein | –4.68 |
| ProA | γ-glutamyl phosphate reductase | –4.72 |
| TolC | Outer membrane protein tolC precursor | –4.92 |
| TalB | Transaldolase | –4.94 |
| NuoC | Bifunctional NADH:ubiquinone oxidoreductase subunit/NADH dehydrogenase | –5.02 |
| CirA | Ferric iron-catecholate outer membrane transporter | –5.21 |
| ProS | Prolyl-tRNA synthetase | –5.32 |
| GcvT | Glycine cleavage system aminomethyltransferase T | –5.82 |
| GlyA | Serine hydroxymethyltransferase | –6.31 |
| GlyS | Glycine tRNA synthetase, β subunit | –6.31 |
| MdoG | Glucan biosynthesis protein G | –7.00 |
| FusA | Elongation factor G | –7.10 |

*Proteins were identified by 2D gel electrophoresis and MALDI-TOF-MS
