# Supplementary material for: Significant Rewiring of the Transcriptome and Proteome of an Escherichia coli Strain Harboring a Tailored Exogenous Global Regulator IrrE
Source: PLoS One. 2012 Jul 5;7(7):e37126. doi: 10.1371/journal.pone.0037126 (PMC3390347; doi:10.1371/journal.pone.0037126)
Supplement: Table S3 — Comparison of changes at the protein and transcriptional levels in strain E1 relative to strain E0. (DOC) [file pone.0037126.s006.doc]

**Table S3.** Comparison of changes at the protein and transcriptional levels in strain E1 relative to strain E0*

| **Gene** | **Fold change at the protein level (Log_2_ ratio)** | **Fold change at the transcriptional level (Log_2_ ratio)** | **Gene** | **Fold change at the protein level (Log_2_ ratio)** | **Fold change at the transcriptional level(Log_2_ ratio)** |
| --- | --- | --- | --- | --- | --- |
| *kdsA* | 7.42 | 0.17 | *crr* | –3.38 | 0.17 |
| *accC* | 6.64 | –1.22 | *lpdA* | –3.51 | –0.10 |
| *pfkA* | 5.38 | 0.89 | *trpS* | –3.57 | –0.10 |
| *nuoF* | 5.08 | 2.54 | *ompX* | –3.64 | –1.09 |
| *gnd* | 5.06 | –0.13 | *pnp* | –3.68 | –0.50 |
| *purH* | 5.06 | –0.72 | *ompT* | –3.85 | –2.42 |
| *rho* | 5.02 | –1.06 | *serC* | –3.9 | –1.16 |
| *pyrG* | 4.64 | –0.54 | *rpsA* | –4.11 | –0.54 |
| *dapA* | 4.64 | –0.07 | *fiu* | –4.11 | –5.07 |
| *pheS* | 4.52 | –1.02 | *aceE* | –4.27 | –1.23 |
| *tnaA* | 4.4 | 5.63 | *tktA* | –4.29 | –0.10 |
| *kdgK* | 4.32 | 0.74 | *oppA* | –4.47 | 1.36 |
| *glpD* | 4.32 | 2.17 | *gapA* | –4.64 | 0.84 |
| *sdhA* | 4.32 | 2.73 | *yhgF* | –4.68 | –1.44 |
| *yncE* | 4.32 | ND | *proA* | –4.72 | –0.09 |
| *lpxA* | 3.39 | –0.53 | *tolC* | –4.92 | –0.06 |
| *ackA* | 3.32 | –1.56 | *talB* | –4.94 | 0.21 |
| *sucD* | 3.24 | 3.09 | *nuoC* | –5.02 | 2.42 |
| *ompW* | 2.56 | 3.82 | *cirA* | –5.21 | –4.48 |
| *rplB* | 2.19 | –0.96 | *proS* | –5.32 | –0.60 |
| *rplI* | 2.06 | –0.53 | *gcvT* | –5.82 | 1.28 |
| *papA* | 1.02 | –0.09 | *glyA* | –6.31 | –0.42 |
| *glpK* | –0.07 | 3.52 | *glyS* | –6.31 | –0.63 |
| *pckA* | –0.89 | 1.73 | *mdoG* | –7.00 | 0.36 |
| *bla* | –2.18 | 0.36 | *fusA* | –7.10 | –0.57 |
| *ompA* | –2.25 | –0.01 |  |  |  |

*Only genes/proteins that were identified using 2D gel electrophoresis and MALDI-TOF-MS are included.
